# Comparative effects of Baduanjin versus brisk walking on postural control and multidimensional functions in early-to-mid-stage Parkinson's Disease: a randomized controlled trial

**DOI:** 10.3389/fneur.2026.1792257

**Published:** 2026-04-22

**Authors:** Yinhang Sun, Panpan Tian, Yibo Xie, Yanjie Guo, Fengtao Liu, Hongxia Xing

**Affiliations:** 1 Department of Neurology, The Third Affiliated Hospital of Xinxiang Medical University, Xinxiang, China; 2 Key Laboratory of Movement Disorders, The Third Affiliated Hospital of Xinxiang Medical University, Xinxiang, Henan, China; 3 Institute of Rehabilitation, Xinxiang Medical University, Xinxiang, Henan, China; 4 Institute of Physical Education, Xinxiang Medical University, Xinxiang, Henan, China

**Keywords:** aerobic exercise, Baduanjin, Parkinson's disease, rehabilitation, wearable devices

## Abstract

**Introduction:**

Baduanjin is a traditional mind-body exercise designed to modulate neural circuits and motor coordination. This study aimed to compare its rehabilitative efficacy against conventional brisk walking on postural control, motor, and non-motor functions in patients with early- to mid-stage Parkinson's disease (PD).

**Methods:**

A 12-week randomized controlled trial was conducted with 32 PD patients (Hoehn and Yahr stages 1–2.5). Participants were randomly assigned to either a Baduanjin group or a Brisk Walking group and underwent 40-min training sessions five times weekly. Primary outcomes included the Movement Disorder Society-Unified Parkinson's Disease Rating Scale Part III (MDS-UPDRS-III) and Berg balance scale (BBS). Secondary outcomes included Montreal Cognitive Assessment (MoCA), Hamilton Anxiety Scale (HAMA), Hamilton Depression Scale (HAMD), Pittsburgh Sleep Quality Index (PSQI), 39-Item Parkinson's Disease Questionnaire (PDQ-39) and objective gait parameters were quantified using wearable sensors during the Timed Up and Go (TUG) test and turning task.

**Results:**

After 12 weeks, the Baduanjin group demonstrated significantly greater improvements than Brisk Walking in MDS-UPDRS-III (*P* < 0.05) and axial subscale improved at 6 weeks (*P* = 0.038) but not 12 weeks (*P* = 0.060). No between-group differences occurred in limb subscale or BBS (all *P* > 0.05).No between-group differences occurred in limb subscale or BBS (all P>0.05). Baduanjin reduced anxiety more at 12 weeks (*P* = 0.014) and improved cognition at 6 weeks (*P* = 0.015). Depression improved within Baduanjin only (*P* = 0.015). Both groups improved sleep and quality of life. Baduanjin showed superior gait parameters including TUG duration, turning time, trunk sagittal angular velocity during sit-to-stand/stand-to-sit; (all *P* < 0.05).

**Conclusion:**

Baduanjin training appears to offer greater improvements in motor symptoms compared to conventional brisk walking in patients with early- to mid-stage Parkinson's disease. Its integration of mindful movement and breathing techniques may particularly address core motor deficits in PD, providing a safe, accessible, and culturally acceptable non-pharmacological rehabilitation option.

**Clinical Trial Registration:**

ChiCTR2300069360 [https://www.chictr.org.cn/].

## 1 Introduction

Parkinson's disease (PD) is a progressive neurodegenerative disorder predominantly affecting middle-aged and elderly populations. Epidemiological studies indicate a global prevalence of approximately 1.6% in individuals aged 65 and older (1–4). Although the exact etiology of PD remains unclear, it is thought to involve a complex interplay of genetic factors (such as mutations in genes like SNCA, LRRK2) and environmental factors (exposure to pesticides, heavy metals) (5). Clinically, the disease is defined by cardinal motor symptoms, including resting tremor, bradykinesia, and rigidity. However, it is the fourth cardinal symptom, postural instability, that often proves to be the most debilitating. This instability progressively impairs balance and functional mobility, posing a major challenge to patients' independence and quality of life (4, 6).

As the disease advances, the motor symptoms of PD can lead to a decline in the patient's activity level and an increased risk of falls (4, 7). It is estimated that 25% of PD patients will experience a hip fracture within 10 years of diagnosis, a complication that leads to significant morbidity, mortality, and socioeconomic burden. Postural instability is recognized as a primary contributor to this elevated fall risk (4). Furthermore, the clinical burden of PD is compounded by a range of non-motor symptoms, such as cognitive impairment, depression, anxiety, and sleep disturbances, all of which severely compromise patients' quality of life (1, 8, 9).

Currently, drug therapy, primarily levodopa-based treatments, remains the mainstay of PD management. While highly effective in the early stages, long-term medication use is frequently complicated by motor fluctuations (e.g., the ‘wearing-off' phenomenon) and dyskinesias. Notably, these therapies offer limited efficacy for axial symptoms, such as postural instability. For medication-refractory cases in advanced stages, deep brain stimulation (DBS) offers a viable surgical option (10). However, as an invasive neuromodulation technique, DBS is associated with potential risks, including infection, hemorrhage, and electrode displacement, and its application is restricted to a select patient population (10, 11). Overall, while current pharmacological and surgical treatments play important roles in PD management, their limitations call for alternative therapeutic strategies. In light of these limitations associated with conventional pharmacological and surgical treatments, there is an increasing exploration of non—pharmacological approaches to manage PD.

Given the limitations of conventional treatments, there is a growing focus on non-pharmacological interventions as complementary therapeutic strategies. Among these, rehabilitation exercises, particularly mind-body therapies like Tai Chi, Qigong, and Baduanjin, have garnered considerable attention (12–14). Baduanjin is a traditional Chinese mind—body exercise, which is characterized by slow, coordinated movements, deep breathing, and mental focus. Preliminary studies suggest that Baduanjin may improve both motor and non-motor symptoms in PD patients, including balance, gait, mood, and sleep (15–17). The holistic nature of Baduanjin, which integrates physical and mental training, appears well-suited to address the multi-systemic manifestations of PD. Its low intensity and accessibility also make it a safe and sustainable option for individuals with mild-to-moderate symptoms (17, 18). Although Dong et al.'s preliminary study suggested short-term benefits of Baduanjin for Parkinson's disease patients (17), its self-controlled design, brief intervention period (3 weeks), and limited objective evaluation measures restricted the generalizability of its conclusions and precluded in-depth exploration of the underlying mechanisms. The present study addresses these limitations by employing a more rigorous randomized controlled trial design, extending the intervention period to 12 weeks, and incorporating objective assessments via wearable devices, thereby providing higher-level evidence for Baduanjin's rehabilitative effects.

Currently, there is a lack of high-quality randomized controlled trials comparing the effects of Baduanjin and conventional aerobic exercise (such as brisk walking) on postural control in patients with Parkinson's disease. Brisk walking was selected as an active comparator as it represents a widely recommended aerobic exercise for PD patients (19, 20), yet its effects on axial and postural symptoms remain unclear. Moreover, many of the published studies are of relatively short duration. Therefore, this study was designed to address this gap by investigating the rehabilitative effects of a 12-week Baduanjin training program on postural control and other functional outcomes in patients with early- to mid-stage PD through a robust RCT design, comparing its efficacy against a conventional Brisk Walking.

## 2 Materials and methods

### 2.1 Participants

From February 2023 to February 2025, we enrolled patients diagnosed with PD who presented to the outpatient clinics or were admitted to the inpatient departments at the Third Affiliated Hospital of Xinxiang Medical University. This study has been approved by the Ethics Committee of the Third Affiliated Hospital of Xinxiang Medical University (Approval Number: K2022-072-01). It has also been registered in the Chinese Clinical Trial Registration Center (ChiCTR) with the registration number: ChiCTR2300069360. The study was performed according to the standards of the 1964 Declaration of Helsinki. All participants gave written informed consent before their inclusion.

### 2.2 Inclusion criteria

(1) Conform to the clinical diagnostic criteria of MDS-PD (2015) (21);
(2) Early PD patients with Hoehn & Yahr (H&Y) stage 1 to 2.5 who can walk independently;

### 2.3 Exclusion criteria

(1) Patients with a history of stroke, head trauma, hydrocephalus, brain tumors, increased intracranial pressure, and intracranial surgery;
(2) Patients with alcohol dependence and central nervous system drug dependence;
(3) There are serious organic lesions of organs such as the heart, liver, and kidneys.

### 2.4 Sample size calculation

An *a priori* power analysis was performed using G^*^Power 3.1 software to estimate the minimum required sample size. We referred to previous studies by Lai (16) and Xiao (20) to determine the appropriate effect size. The statistical test was specified as ANOVA: Repeated measures, within-between interaction with F tests as the test family, a configuration consistent with the generalized estimating equation (GEE) model adopted for primary outcome analysis in the present study. Based on previous Baduanjin and exercise trials in Parkinson's disease, a medium effect size (*f* = 0.30) was assumed, with Type I error probability (α) set to 0.05 (two-tailed) and a minimum power level of 0.95, yielding an estimated required sample size of 32 participants (16 per group). Consequently, 38 participants (19 per group) were randomized to account for a potential 15–20% attrition rate. Ultimately, 32 participants (16 per group) completed the trial and were included in the per-protocol (PP) analysis, meeting the sample size requirement estimated by the *a priori* analysis. Furthermore, *post-hoc* power analysis based on the large observed effect size (*f* = 0.46) for the primary outcome (MDS-UPDRS-III) confirmed that achieved statistical power exceeded 95% for both the intention-to-treat (ITT, *n* = 38) and Per protocol (PP, *n* = 32) populations, affirming the adequacy of the sample size to detect significant intervention effects.

### 2.5 Randomization and masking

**Figure 1:**
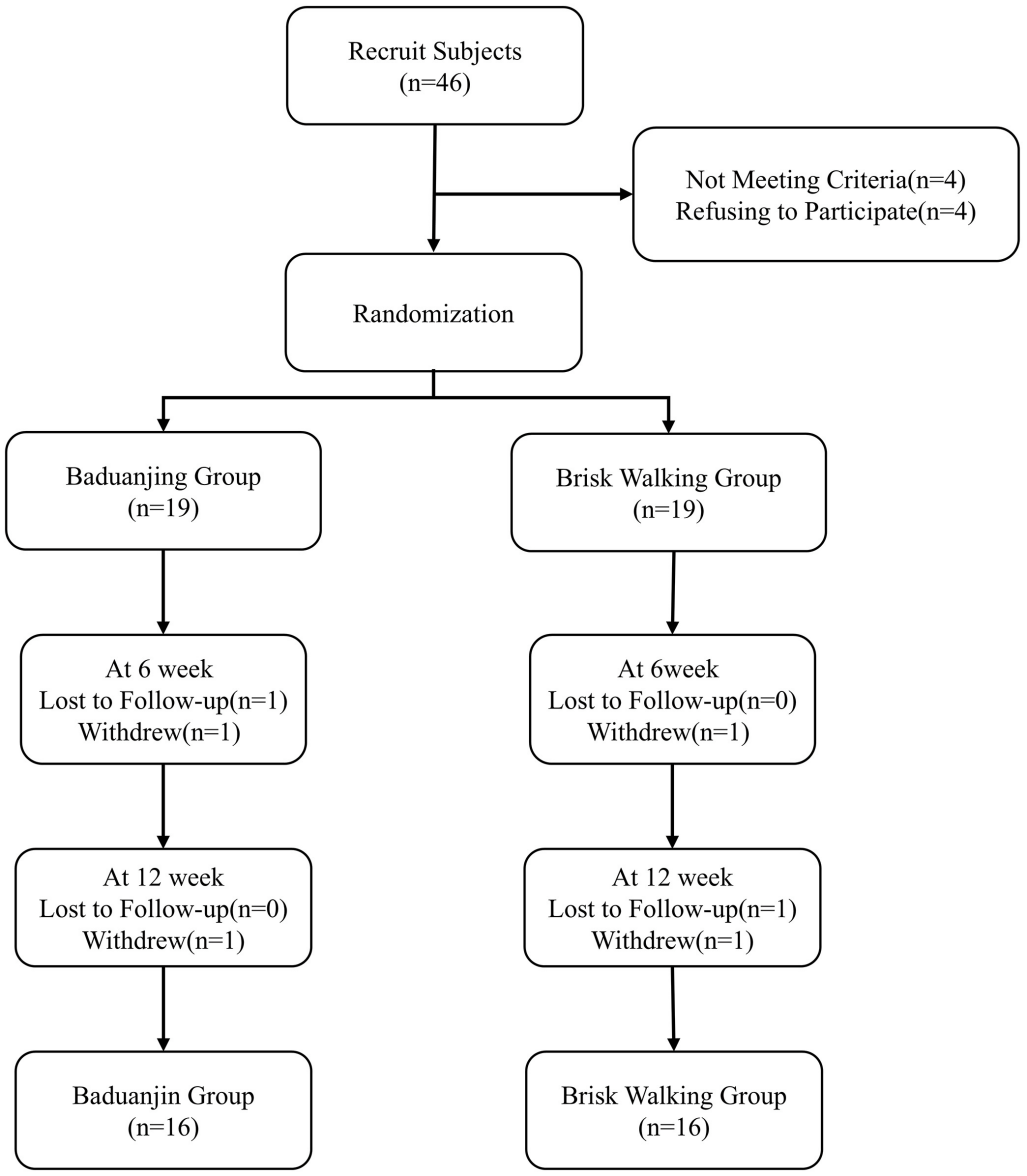
Consort participant flow diagram.

Participants were randomly assigned to Baduanjin or Brisk Walking in a 1:1 ratio using computer-generated random numbers (IBM SPSS Statistics 25.0). Allocation concealment was achieved using sequentially numbered, opaque, sealed envelopes prepared by an independent statistician. Envelopes were opened by the study coordinator only at enrollment. A single-blind design was implemented with outcome assessors blinded to group allocation. Assessors were independent research staff with no involvement in intervention delivery, and assessments were conducted in a separate room. Participants were instructed not to disclose their group assignment. Assessor blinding success was verified at study completion by asking assessors to guess group allocations (Figure 1).

### 2.6 Procedures

Throughout the 12-week study period, all participants maintained their pre-existing medication regimens. Participants in both the Baduanjin (Supplementary Material Figure 1) and Brisk Walking groups adhered to a structured exercise protocol of 40 min per session, five times per week. Each session comprised an 8-min warm-up, a 24-min core training component, and an 8-min cool-down period. To ensure comparable exercise loads between groups, intensity was standardized and monitored in real-time using the Borg Rating of Perceived Exertion (RPE) 6–20 scale, with a target range of 11–13 (corresponding to approximately 40%−60% of Heart Rate Reserve, HRR). This intensity range is consistent with previous studies characterizing Baduanjin and similar mind-body exercises as low-to-moderate intensity activities, typically eliciting 50%−55% of maximum heart rate (22). Participants were instructed to self-measure their heart rate and blood pressure after each training session using automated upper-arm blood pressure monitors to verify adherence to the target intensity zone and monitor safety. Research staff reviewed training logs weekly to identify any concerning patterns. The core training for the Baduanjin group consisted of the standardized routine prescribed by the General Administration of Sport of China. To ensure intervention fidelity, certified instructors provided standardized, in-person training prior to the intervention, supplemented by illustrated manuals and video tutorials. The Brisk Walking group's core training involved walking at a maximal controllable pace with vigorous arm swings. A rehabilitation physician conducted regular follow-ups with all participants to assess clinical progression and monitor for adverse events. During the intervention, adherence was monitored via daily training logs and remote video supervision.

### 2.7 Outcome measures

Demographic and baseline clinical characteristics, including age, gender, years of education, and disease duration, were recorded for all participants. A comprehensive battery of clinical assessments was administered at baseline, 6 weeks, and 12 weeks post-intervention. To minimize the confounding effects of motor fluctuations, all assessments at each time point were systematically conducted while participants were in their “on-period” state (approximately 2 h post-dose).

#### 2.7.1 Primary outcome measures

Motor symptoms were rated with the Movement Disorder Society-Unified Parkinson's Disease Rating Scale Part III (MDS-UPDRS-III) (23), with additional sub-scores for (1) axial signs (items 3.9–3.14) and (2) limb-related items (items 3.3b–e and 3.4–3.8). Balance function was evaluated using the Berg Balance Scale (BBS)(24).

#### 2.7.2 Secondary outcome measures

The secondary outcome battery encompassed cognitive and non-motor domains: cognitive function was assessed with the Montreal Cognitive Assessment (MoCA) (25); mood was quantified using the Hamilton Depression Scale (HAMD) and the Hamilton Anxiety Scale (HAMA); sleep disturbances were measured by the Pittsburgh Sleep Quality Index (PSQI); and disease-specific quality of life was captured with the 39-Item Parkinson's Disease Questionnaire (PDQ-39)(26).

##### 2.7.2.1 Gait analysis

Quantitative analysis of gait and postural stability was performed at baseline and 12 weeks post-intervention using a wearable motion analysis system (GYENNO Science & Technology Co., Ltd.) (27). The system comprised 10 wireless inertial measurement units (IMUs), securely affixed to the sternum, lumbar spine, bilateral wrists, thighs, shanks, and feet. This configuration was designed for multi-dimensional quantification of postural control, with the sternal sensor monitoring trunk pitch and roll, the lumbar sensor capturing trunk rotation and lateral flexion, and lower limb sensors recording parameters related to support and gait dynamics (28, 29). The assessment protocol comprised two standardized motor tasks: the 5-meter Timed Up and Go (TUG) test (30) and a 360-degree in-place turning task (31). During the TUG test, participants were instructed to rise from a standard armchair, walk 5 meters at their self-selected usual pace, execute a 180-degree turn, walk back to the chair, and sit down. For the turning task, participants performed two complete, in-place turns sequentially in both clockwise and counterclockwise directions within a marked 0.7 m × 0.7 m area. For both tasks, the total duration and relevant kinematic parameters were recorded for analysis.

### 2.8 Statistical analysis

The normality of data distribution was first assessed using the Shapiro-Wilk test. Normally distributed continuous variables were presented as mean ± standard deviation (SD), while non-normally distributed data were presented as median (P25, P75). Categorical variables were summarized as frequencies and percentages (*n*, %). Baseline demographic and clinical characteristics were compared between groups using independent *t*-tests for normally distributed data, Mann-Whitney U tests for non-normally distributed data, and Pearson's chi-square (χ^2^) tests for categorical data. **For the primary outcomes, missing data at 6 weeks and 12 weeks post-intervention were handled using multiple imputation by chained equations. The imputation model included baseline covariates, treatment group assignment, time, and all observed outcome values. The intervention effects on the primary outcome measures [MDS-UPDRS-III total score, axial sub-scores (items 3.9–3.14), limb sub-scores (items 3.3b–e and 3.4–3.8), and Berg Balance Scale (BBS) score] were primarily evaluated using intention-to-treat (ITT) analysis, with PP analysis performed as a sensitivity analysis. Secondary outcome measures were primarily assessed in the PP set. GEE models, adjusted for baseline values as a covariate, were used to examine the intervention effects. The models included the main effects of treatment group, time (categorical), baseline outcome, and the group** **×** **time interaction as the primary effect of interest. For gait parameters, between-group differences were evaluated using baseline-adjusted ANCOVA. Normality was assessed on model residuals; non-normally distributed parameters were analyzed using Mann-Whitney U tests**. A two-tailed *P-value* < 0.05 was considered statistically significant for all analyses. All statistical analyses were performed using SPSS Statistics for Windows, Version 25.0 (IBM).

## 3 Results

### 3.1 Baseline characteristics of the research subjects

A total of 46 participants were enrolled in the study. After screening and randomization, there were 19 participants each in the Baduanjin group and the Brisk Walking group. After 12 weeks of intervention, 16 participants in each group completed the entire study and were included in the statistical analysis. There were no statistically significant differences between the Baduanjin and Brisk Walking groups at baseline in any demographic or clinical characteristics, including age, gender, disease duration, education level, H&Y stage, and levodopa equivalent daily dose (LEDD). Furthermore, all baseline clinical assessments—including the MDS-UPDRS-III, BBS, MoCA, HAMD, HAMA, PSQI, and PDQ-39—were comparable between groups (all *P* > 0.05), confirming the homogeneity of the participants prior to the intervention. A detailed summary of baseline characteristics is provided in (Table 1). No adverse events were reported during the 12-week intervention period in either group. Specifically, no falls, injuries, or exercise-related discomfort occurred.

**Table 1:** Demographic characteristics of participants in different groups.

Group	Baduanjin group (*n* = 16)	Brisk walking group (*n* = 16)	*P* value
Age (years)	66.12 (6.94)	64.38 (9.90)	0.567
Gender (M/F)	9/7	10/6	0.718
Disease duration (years)	5.59 (3.09)	5.29 (3.56)	0.795
Education level	10.31 (2.39)	9.44 (2.71)	0.340
H&Y stage	2.00 (1.00, 2.50)	2.00 (2.00, 2.50)	0.282
LEDD	532.00 (190.39)	484.50 (225.59)	0.525
MDS-UPDRS-III	40.06 (13.19)	43.75 (11.39)	0.404
BBS	50.56 (5.51)	48.69 (6.35)	0.380
MOCA	19.73 (4.10)	19.69 (3.77)	0.974
HAMD	3.50 (1.00,9.00)	5.50 (4.00,12.50)	0.197
HAMA	9.63 (6.89)	9.50 (6.86)	0.959
PSQI	7.31 (4.73)	8.13 (4.41)	0.619
PDQ-39	13.50 (8.63)	13.06 (8.03)	0.883

Normally distributed continuous variables are presented as mean (SD); non-normally distributed variables as median (P25, P75). H&Y, Hoehn-Yahr; LEDD, levodopa equivalent daily dosage; MDS-UPDRS-III, Movement Disorder Society-Unified Parkinson's Disease Rating Scale Part III; BBS, Berg Balance Scale; MOCA, Montreal Cognitive Assessment; HAMD, Hamilton Depression Scale; HAMA, Hamilton Anxiety Rating Scale; PSQI, Pittsburgh Sleep Quality Index; PDQ – 39, 39-Item Parkinson's Disease Questionnaire.

### 3.2 Effects of interventions on clinical outcomes

#### 3.2.1 Motor function

**Figure 2:**
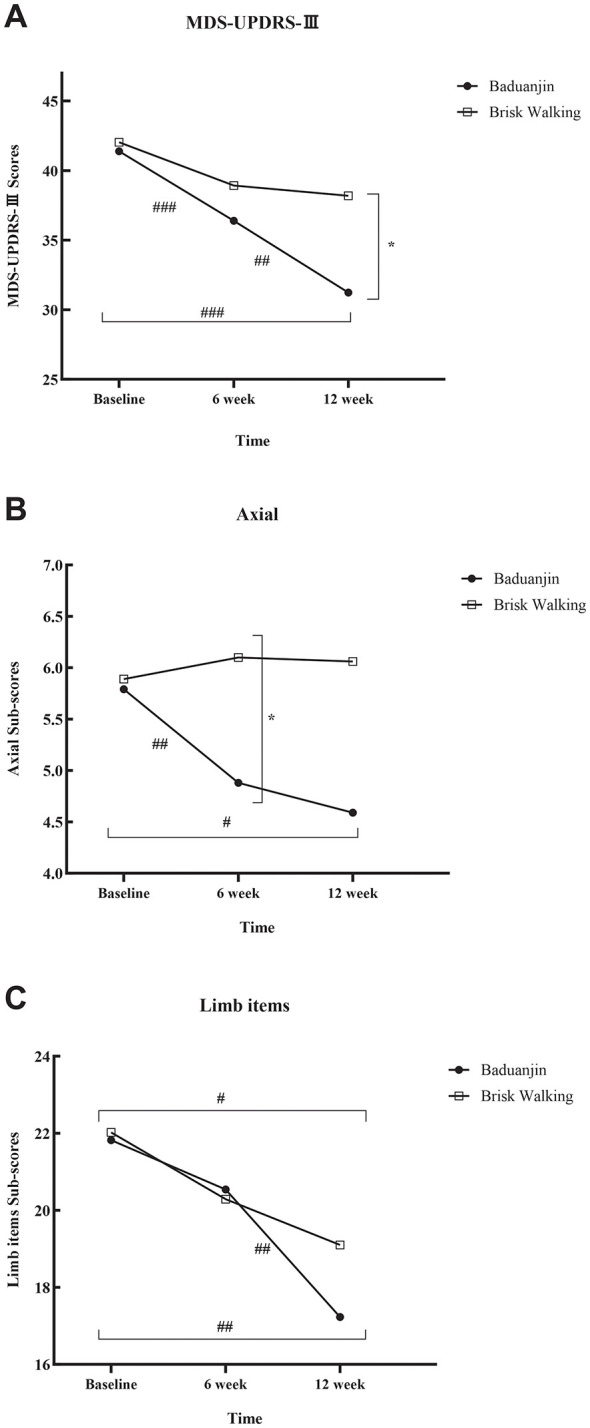
Longitudinal changes in Primary outcomes over 12 weeks. **(A)** Total score of MDS-UPDRS-III; **(B)** The score of Axial; **(C)** The score of Limb items. Between-group comparisons: ^*^P < 0.05; Within-group pairwise comparisons across time points: ^#^ *P* < 0.05, ^##^ *P* < 0.01, ^###^ *P* < 0.001.

Baduanjin training resulted in greater motor improvement than brisk walking, as measured by the MDS-UPDRS-III. GEE analysis revealed a significant Group × Time interaction (*P* = 0.041). *Post-hoc* analyses showed that while both groups improved over time, the Baduanjin group demonstrated significantly greater reduction in scores compared to the brisk walking group at the 12-week intervention assessment (mean difference,−6.95 [95% CI,−11.79 to−2.11]; *P* = 0.005), with a large between-group effect size (Cohen's d = 0.92). A numerical advantage favoring Baduanjin was also observed at the 6-week intervention assessment, although this difference did not reach statistical significance (mean difference,−2.53 [95% CI,−6.56 to 1.50]; *P* = 0.219). Results from the PP analysis were consistent with these ITT findings (Figure 2A, Table 2).

**Table 2:** Comparison of primary outcomes between baduanjin and brisk walking groups^a^.

Variable	Study group, Mean (SE)			*P* value		
	**Baduanjin group**	**Brisk walking group**	**Mean difference (95% CI)**	**Group effect**	**Time effect**	**Interaction effect**
MDS-UPDRS-III						
ITT	19	19				
Baseline	41.39 (0.548)	42.03 (0.463)	−0.64 (-2.06–0.79)			
6 week	36.39 (1.271)	38.92 (1.624)	−2.53 (-6.56–1.50)	0.010^*^	0.000^***^	0.041^*^
12 week	31.23 (1.401)	38.19 (2.016)	−6.95 (-11.79–−2.11)			
Per-Protocol	16	16				
Baseline	41.54 (0.654)	42.27 (0.550)	−0.74 (-2.44–0.97)			
6 week	37.48 (0.988)	39.40 (1.884)	−1.92 (-6.08–2.23)	0.018^*^	0.000^***^	0.016^*^
12 week	30.48 (1.562)	38.34 (2.336)	−7.86 (-13.41–−2.31)			
BBS						
ITT	19	19				
Baseline	50.42 (1.127)	48.74 (1.297)	1.68 (-1.68–5.05)			
6 week	50.37 (1.007)	48.53 (1.363)	1.84 (-1.48–5.16)	0.267	0.899	0.977
12 week	50.53 (1.002)	48.95 (1.224)	1.58 (-1.52–4.68)			
Per-Protocol						
Baseline	49.77 (0.208)	49.48 (0.260)	0.30 (-0.37–0.97)			
6 week	49.71 (0.572)	48.98 (0.949)	0.73 (-1.44–2.91)	0.552	0.733	0.867
12 week	49.90 (0.950)	49.91 (0.663)	−0.02 (-2.26–2.23)			
Axial						
ITT	19	19				
Baseline	5.79 (0.087)	5.89 (0.170)	−0.10(-0.48–0.27)			
6 week	4.88 (0.292)	6.10 (0.513)	−1.22(-2.38–−0.06)	0.013^*^	0.373	0.130
12 week	4.59 (0.462)	6.06 (0.632)	−1.47(-3.01–0.06)			
PP	16	16				
Baseline	5.75 (0.097)	5.75 (0.156)	0.00 (-0.36–0.36)			
6 week	5.12 (0.281)	5.81 (0.449)	−0.69 (-1.72–0.35)	0.042^*^	0.235	0.140
12 week	4.12 (0.384)	5.87 (0.746)	−1.75 (-3.39–−0.11)			
limb items						
ITT	19	19				
Baseline	21.82 (0.557)	22.02 (0.446)	−0.21 (-1.61–1.20)			
6 week	20.54 (0.898)	20.29 (1.060)	0.25 (-2.46–2.97)	0.481	0.000^***^	0.370
12 week	17.23 (1.136)	19.10 (1.258)	−1.87 (-5.18–1.44)			
PP
Baseline	22.06 (0.633)	22.63 (0.440)	−0.57 (-2.10–0.97)			
6 week	21.19 (0.908)	19.81 (1.149)	1.37 (-1.48–4.23)	0.617	0.001^**^	0.061
12 week	17.12 (1.269)	19.38 (1.466)	−2.25 (-6.02–1.51)			

^a^A generalized estimated equation model, with baseline measurement as the covariate, was used to analyze the data.

ITT, intention to treat; PP, Per protocol; MDS-UPDRS-III, Movement disorder society-unified parkinson's disease rating scale part III; BBS, berg balance scale.

^*^P < 0.05, ^**^ P < 0.01, ^***^P < 0.001.

For the axial subscale of MDS-UPDRS-III, ITT analysis showed that the Baduanjin group exhibited significantly greater scores reductions compared with the brisk walking group at the 6-week intervention (mean difference, −1.22 [95% CI, −2.38 to −0.06]; *P* = 0.038). A similar numerical advantage favoring Baduanjin was observed at 12 weeks, though this difference did not reach conventional statistical significance (mean difference, −1.47 [95% CI, −3.01 to 0.06]; *P* = 0.060). The overall between-group effect size was moderate (Cohen's d = 0.61). GEE analysis showed no significant Group × Time interaction effect (*P* = 0.130). Similar findings were also observed in the PP analysis. Notably, in the PP analysis restricted to study completers, the between-group difference at 12 weeks reached statistical significance (*P* = 0.037) (Figure 2B, Table 2).

For the limb subscale scores of MDS-UPDRS-III, no significant between-group differences were observed at the 6-week (mean difference, 0.25 [95% CI, −2.46 to 2.97]; *P* = 0.481) or 12-week assessment (mean difference, −1.87 [95% CI, −5.18 to 1.44]; *P* = 0.481). The overall between-group effect size was small (Cohen's d = 0.36). GEE analysis showed no significant Group × Time interaction effect (*P* = 0.370). Similar findings were also observed in the PP analysis (Figure 2C, Table 2).

No significant between-group differences in BBS scores were detected between the two groups at 6-week intervention (mean difference, 1.84 [95% CI, −1.48 to 5.16]; *P* = 0.267) or 12-week intervention (mean difference, 1.58 [95% CI, −1.52 to 4.68]; *P* = 0.267), and no significant Group × Time interaction effect was found (*P* = 0.977). A significant overall time effect was observed for both groups, confirming that both exercise modalities improve static balance relative to baseline. Similar findings were also observed in the PP analysis (Table 2).

#### 3.2.2 Cognitive function

**Figure 3:**
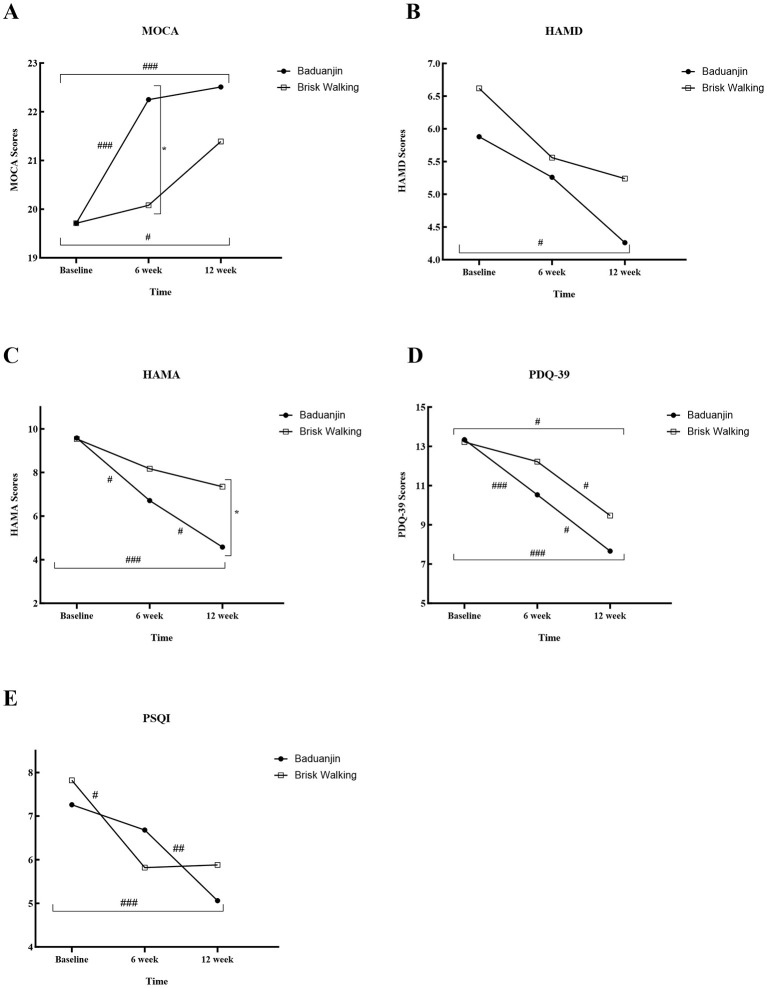
Longitudinal changes in Secondary outcomes over 12 weeks. **(A)** MOCA: Montreal Cognitive Assessment; **(B)** HAMD: Hamilton Depression Scale; **(C)** HAMA: Hamilton Anxiety Rating Scale; **(D)** PDQ – 39: 39-Item Parkinson's Disease Questionnaire; **(E)** PSQI: Pittsburgh Sleep Quality Index. Between-group comparisons: ^*^P < 0.05; Within-group pairwise comparisons across time points: ^#^ *P* < 0.05, ^##^ *P* < 0.01, ^###^ *P* < 0.001.

For the MoCA score, the analysis identified significant main effects for Time (*P* ≤ 0.001) and Group (*P* = 0.044), but the Group × Time interaction was not significant. *Post-hoc* tests showed that the Baduanjin group's scores was significantly higher than that of the Brisk Walking group at the 6-week timepoint (mean difference, 2.17; 95% CI, 0.42 to 3.91; *P* = 0.015). Both groups showed significant within-group improvements at 6 and 12 weeks compared to baseline (Table 3, Figure 3A).

**Table 3:** Comparison of secondary outcomes between baduanjin and brisk walking groups^a^.

Variable	Study group, Mean (SE)			***P*** value		
	**Baduanjin group (*****n*** = **16)**	**Brisk walking group (*****n*** = **16)**	**Mean difference (95% CI)**	**Group effect**	**Time effect**	**Interaction effect**
MOCA						
Baseline	19.71 (0.163)	19.71 (0.146)	0.01 (-0.42–0.44)	0.044^*^	0.000^***^	0.074
6 week	22.25 (0.579)	20.08 (0.673)	2.17 (0.42–3.91)			
12 week	22.51 (0.717)	21.39 (0.796)	1.12 (−0.98–3.22)			
HAMD						
Baseline	5.88 (0.392)	6.62 (0.431)	−0.74 (−1.95–0.47)	0.245	0.117	0.851
6 week	5.26 (0.393)	5.56 (0.980)	−0.30 (−2.35–1.74)			
12 week	4.26 (0.439)	5.24 (1.081)	−0.99 (−3.25–1.27)			
HAMA						
Baseline	9.58 (0.601)	9.54 (0.597)	0.04 (−1.61–1.70)	0.036^*^	0.000^***^	0.218
6 week	6.71 (1.048)	8.17 (0.964)	−1.46 (−4.24–1.33)			
12 week	4.58 (0.713)	7.35 (0.879)	−2.77 (−4.98– −0.55)			
PSQI						
Baseline	7.62 (0.291)	7.82 (0.264)	−0.20 (−0.98–0.57)	0.917	0.001^**^	0.129
6 week	6.68 (0.454)	5.82 (0.705)	0.86 (−0.79–2.51)			
12 week	5.06 (0.480)	5.88 (0.917)	−0.83 (−2.86–1.20)			
PDQ-39						
Baseline	13.34 (0.602)	13.22 (0.559)	0.13 (−1.48–1.73)	0.238	0.000^***^	0.610
6 week	10.53 (0.815)	12.22 (1.617)	−1.69 (−5.24–1.86)			
12 week	7.66 (1.159)	9.47 (1.166)	−1.81 (−5.04–1.42)			

^a^A generalized estimated equation model, with baseline measurement as the covariate, was used to analyze the data.

MOCA, Montreal cognitive assessment; HAMD, Hamilton depression scale; HAMA, Hamilton anxiety rating scale; PSQI, Pittsburgh sleep quality index; PDQ – 39, 39-Item parkinson's disease questionnaire.

^*^P < 0.05, ^**^ P < 0.01, ^***^P < 0.001.

#### 3.2.3 Mood

For anxiety (HAMA), the GEE model revealed significant main effects of Time (*P* ≤ 0.001) and Group (*P* = 0.036). *Post-hoc* analyses showed a progressive and significant reduction in HAMA scores within the Baduanjin group from baseline to 6 weeks (mean difference,−2.88; 95% CI,−5.29 to−0.46; *P* = 0.020) and to 12 weeks (mean difference,−5.00; 95% CI,−7.15 to−2.85; *P* ≤ 0.001). Consequently, at 12 weeks, the HAMA scores of the Baduanjin group were significantly lower than those of the Brisk Walking group (mean difference,−2.77; 95% CI,−4.98 to−0.55; *P* = 0.014). For depression (HAMD), a significant within-group improvement from baseline was observed exclusively in the Baduanjin group at 12 weeks (mean difference,−1.63; 95% CI,−2.93 to−0.32; *P* = 0.015) (Table 3, Figures 3B–C).

#### 3.2.4 Sleep and Quality of Life

For both PSQI and PDQ-39 scores, a significant main effect of Time was found *(P* ≤ 0.001 for both). However, no significant Group or Group × Time interaction effects were detected (all *P* > 0.05), indicating that both interventions yielded improvements over time. Subsequent within-group analyses for sleep quality showed that the Baduanjin group achieved a significant reduction in PSQI scores at 12 weeks (mean difference,−2.56; 95% CI,−3.77 to−1.35; *P* ≤ 0.001), with the improvement from week 6 to 12 also being significant (mean difference,−1.63; 95% CI,−2.84 to−0.41; *P* = 0.009). The Brisk Walking group showed a significant reduction at 6 weeks (mean difference,−2.00; 95% CI,−3.57 to−0.43; *P* = 0.012). For quality of life, the Baduanjin group reported significant reductions in PDQ-39 scores at 6 weeks (mean difference,−2.81; 95% CI,−4.15 to−1.48; *P* < 0.001) and 12 weeks (mean difference,−5.69; 95% CI,−8.86 to−2.51; *P* ≤ 0.001). The Brisk Walking group also showed significant reductions at 12 weeks (mean difference,−3.75; 95% CI,−6.60 to−0.90; *P* = 0.010) (Table 3, Figures 3D–E).

### 3.3 Analysis of the intervention effect on gait stability

Following the 12-week intervention, the Baduanjin group demonstrated significantly improved performance in multiple gait parameters compared with the brisk walking group. Significant differences were observed in TUG duration (*F* = 14.15, *P* = 0.001, partial η^2^ = 0.328), Standing up-Trunk - Max Sagittal Angular Velocity (*U*= 192.0, *P* = 0.046, Cohen's d = 0.75), Sitting down-Trunk - Max Sagittal Angular Velocity (*F* = 9.61*, P* = 0.004, partial η^2^ = 0.249), Turning - Average Duration (*U* = 81.0, *P* = 0.030, Cohen's d = 0.80), Left -Turning Duration (*U* = 61.0, *P* = 0.012, Cohen's d = 0.97), and Turning - Peak Velocity (*F* = 5.87, *P* = 0.021, partial η^2^ = 0.155). No significant between-group differences were found for Right-Turning Duration and Turning - Average Angular Velocity (all *P* > 0.05) (Table 4).

**Table 4:** Within- and between-group comparisons of gait parameters from baseline to post-intervention.

Variable	Study group		*P* value
	**Baduanjin group (*****n*** = **16)**	**Brisk walking group (*****n*** = **16)**	
TUG (sec)			
Baseline	17.56 (4.92)	17.78 (4.03)	0.892
12 week	13.88 (4.46)	16.85 (3.12)	0.001^**^
Standing up-trunk–max sagittal angular velocity (degree/sec)			
Baseline	58.13 (47.17, 63.76)	51.47 (46.70, 64.02)	0.853
12 week	66.54 (56.86, 73.83)	57.15 (48.94, 63.84)	0.046^*^
Sitting down-trunk–max sagittal angular velocity (degree/sec)			
Baseline	53.14 (14.70)	50.77 (16.49)	0.670
12 week	74.27 (18.21)	55.14 (19.08)	0.004^**^
Turning–average duration (sec)			
Baseline	10.70 (8.15, 14.85)	10.79 (9.20, 12.70)	0.805
12 week	8.64 (7.11, 11.65)	12.27 (9.09, 13.54)	0.030^*^
Left-turning duration (sec)			
Baseline	9.91 (8.32, 16.46)	12.12 (10.69, 12.86)	0.820
12 week	8.07 (6.91, 12.68)	12.98 (9.92, 14.92)	0.012^*^
Right-turning duration (sec)			
Baseline	10.14 (7.83, 14.03)	9.53 (7.41, 12.29)	0.769
12 week	8.64 (7.49, 10.43)	11.78 (7.94, 13.34)	0.061
Turning–peak velocity (degree/sec)			
Baseline	135.04 (33.27)	134.77 (40.35)	0.983
12 week	150.43 (29.95)	129.68 (44.41)	0.021^*^
Turning–average angular velocity (degree)			
Baseline	74.87 (27.97)	74.80 (25.80)	0.993
12 week	80.83 (23.20)	70.35 (23.84)	0.063

Normally distributed continuous variables are presented as mean (SD); non-normally distributed variables as median (P25, P75).

Asterisks denote significant between-group differences at the same time point: ^*^P < 0.05, ^**^P < 0.01.

TUG, Timed Up and Go.

## 4 Discussion

This 12-week randomized controlled trial systematically evaluated the rehabilitation efficacy of Baduanjin training in patients with early- to mid-stage Parkinson's disease. We assessed motor function, cognition, emotion, sleep, and quality of life using standardized clinical scales, and employed wearable sensor technology to objectively quantify dynamic postural stability during functional mobility tasks. Our findings demonstrate that Baduanjin training yields superior improvements in motor function (MDS-UPDRS-III) and objective postural stability compared to conventional brisk walking in PD patients, particularly in dynamic balance control during functional mobility tasks. These results provide objective evidence supporting Baduanjin as a targeted intervention for motor symptoms in Parkinson's disease rehabilitation.

This study demonstrates that a 12-week Baduanjin intervention significantly improves core motor symptoms and axial motor function in patients with PD, with greater reductions in MDS-UPDRS-III total scores and axial sub-scores compared to the brisk walking group. Objective wearable sensor data further revealed shortened TUG times, increased maximum sagittal trunk angular velocity during sit-to-stand transitions, and reduced turning duration in the Baduanjin group. These findings indicate enhanced motor initiation, movement transition efficiency, and trunk control agility—axial impairments that are particularly refractory to dopaminergic therapy and challenging in PD (32, 33).

The benefits likely stem from Baduanjin's distinctive slow, coherent movements, emphasizing repeated center-of-gravity shifts, single-leg support, and trunk rotations. These elements provide task-specific training for vestibular-proprioceptive integration and core muscle coordination, compensating for basal ganglia dysfunction-induced sensory-motor deficits (34, 35)and improving postural stability. This mechanism aligns with Tai Chi's effects on gait biomechanics, such as reduced center-of-mass excursions and center-of-pressure separation (36, 37). Notably, the overall motor improvement showed a large effect size (MDS-UPDRS-III: Cohen's d = 0.92), and the effect size for axial symptoms (Cohen's d = 0.61) was larger than that for limb symptoms (Cohen's d = 0.36), suggesting a relatively greater benefit for axial dysfunction. This may reflect the task-specific nature of Baduanjin's axial-targeted training components, highlighting its potential value as a complementary intervention for axial impairment in PD. In contrast, brisk walking primarily provides linear aerobic and limb activation benefits, offering a more stringent active control. Compared with the pooled mean difference of−5.37 points reported in the meta-analysis by Lai et al. (15), the present study observed a larger effect size. This discrepancy may be explained by the longer intervention duration (12 weeks) and the use of intensity-matched brisk walking as an active comparator rather than usual care or no intervention. Additionally, the between-group difference of approximately 10 points in MDS-UPDRS-III scores exceeded the reported minimal clinically important difference (MCID = 2.5 points) (38), supporting clinically meaningful motor benefits. Although Dong et al. (17) previously suggested short-term benefits of Baduanjin, the current randomized controlled trial design, extended duration, and incorporation of objective wearable assessments provide higher-level evidence for its rehabilitative effects.

The results of this study indicate that 12 weeks of Baduanjin training can improve non-motor symptoms in patients with PD. At the end of the intervention, the Baduanjin group showed significantly higher MoCA scores and significantly lower HAMA scores compared with the brisk walking group. In addition, only the Baduanjin group exhibited a significant within-group improvement in HAMD scores. These findings are consistent with previous studies suggesting that mind-body exercises, such as Tai Chi and yoga, can effectively improve emotional and cognitive function in patients with PD (39, 40). The Baduanjin group demonstrated more notable improvements in anxiety symptoms (HAMA) and a significant within-group reduction in depressive symptoms (HAMD). This pattern suggests that simple aerobic exercise alone (such as brisk walking) may be insufficient to markedly improve depressive symptoms in PD patients, whereas the unique characteristics of Baduanjin may play a contributing role. Baduanjin integrates regulated deep breathing with mindful movement, which may modulate autonomic nervous system function by enhancing parasympathetic activity and reducing physiological arousal (41, 42); Furthermore, the complex movement sequences in Baduanjin require learning, memorization, and continuous execution, constituting a form of cognitive-motor dual-task training that may engage executive function and promote neural plasticity (43). However, these interpretations remain speculative, as the present study did not include direct measures of autonomic function, brain-derived neurotrophic factor levels, or neuroimaging. Future studies incorporating mechanistic assessments are needed to test these hypotheses.

The direct comparison in our study highlights potential advantages of Baduanjin as a multimodal intervention over brisk walking for PD management. Both interventions improved patients' quality of life (PDQ-39). Brisk walking, as a recognized aerobic exercise, offers clear cardiovascular and metabolic benefits (44). However, it did not produce statistically significant improvements in fine motor skills, balance ability, or certain cognitive-emotional domains. In contrast, Baduanjin demonstrated clear advantages in the aforementioned areas. Baduanjin incorporates several integrated elements that distinguish it from conventional aerobic exercise. These include slow, controlled movements for balance training, varied postures for enhancing flexibility, and focused attention on coordinated movement-breath sequences (45). These components engage both cognitive and autonomic regulatory systems, potentially addressing motor and non-motor symptoms synergistically through the aforementioned physiological and psychological mechanisms. This corresponds to the reports on the neuroprotective effect of Tai Chi in delaying the degeneration of the substantia nigra striatum, the sensory reweighting effect (46), in enhancing proprioceptive input to compensate for visual defects (42), and the cognitive-motor dual-task training effect in improving the integration ability of executive function and posture control (34). Long-term persistence in Baduanjin may bring deeper benefits beyond functional improvement. However, given the relatively short intervention duration and modest sample size, these preliminary findings warrant validation in larger, longer-term studies to determine whether Baduanjin can produce sustained neuroprotective effects and functional improvements in PD patients.

Although previous studies have reported that Baduanjin can improve balance function in patients with Parkinson's disease (15), the present study found no significant between- or within-group differences in BBS scores. This lack of difference is likely attributable, at least in part, to a ceiling effect of the BBS in early-to-mid-stage PD patients. Supporting this interpretation, baseline BBS scores in our sample were relatively high (Baduanjin group: 50.56 ± 5.51; brisk walking group: 48.69 ± 6.35), with many participants scoring near or at the maximum. Previous research has similarly shown that the BBS has limited sensitivity for detecting subtle balance deficits or improvements in higher-functioning, early-stage PD populations (37, 38, 47, 48). In contrast, the instrumented gait assessments (TUG duration, turning duration, and trunk sagittal angular velocity) successfully detected significant between-group differences favoring Baduanjin. These findings suggest that objective, quantitative movement measures may offer greater sensitivity than traditional clinical scales such as the BBS when evaluating balance and axial motor function in patients with relatively preserved baseline performance.

This study also has some limitations. First, the small sample size may compromise statistical power and generalizability; multicenter trials with larger cohorts are needed for validation. Second, the sample had a higher proportion of males (59.4% vs. 40.6% female). Although consistent with PD epidemiology, sex-specific responses to exercise warrant further investigation with more balanced cohorts. Third, participants were restricted to early/mid-stage PD. As advanced PD involves distinct pathophysiology—such as severe postural instability and multimorbidity—the efficacy and safety of Baduanjin in this population require further study. Fourth, although the 12-week intervention demonstrated short-term benefits, the absence of long-term follow-up (e.g., 3, 6, or 12 months post-intervention) precludes assessment of the sustainability of these effects and potential disease-modifying benefits. Extended follow-up periods are essential in future studies to determine whether the observed improvements persist after intervention cessation. Fifth, although the completion rate was 84.2% (32/38) with exercise logs maintained, session-by-session attendance was not systematically tracked. Finally, we focused solely on clinical outcomes. Future work should investigate underlying neural mechanisms using advanced neuroimaging (e.g., fMRI for functional connectivity), neurochemistry (e.g., MRS for neurotransmitter metabolism), and neuromuscular analyses (e.g., EMG for motor control).

## Data Availability

The original contributions presented in the study are included in the article/Supplementary material, further inquiries can be directed to the corresponding authors.
