# Supplementary material for: Comparative effects of Baduanjin versus brisk walking on postural control and multidimensional functions in early-to-mid-stage Parkinson's Disease: a randomized controlled trial
Source: Front Neurol. 2026 Apr 22;17:1792257. doi: 10.3389/fneur.2026.1792257 (PMC13143543; doi:10.3389/fneur.2026.1792257)
Supplement: Supplementary file 1 [file Data_Sheet_1.docx]

Supplementary Material

# Figure 1. Baduanjin Exercises

A: Hold both hands up to regulate the triple energizer: Breathe in while raising both hands above the head with the palms facing upward. Hold your breath for two seconds when fully stretched. Exhale when returning to the center. Repeat six times.

B: Bending the bow left and right like shooting an eagle: Bend your knees to do a lower horse stance. Open your arms to the left, as if drawing a bow and releasing an arrow. Return to the center and repeat three times on each side.

C: Regulating the spleen and stomach requires lifting one arm alone: Press the hands in opposite directions, one above the head with the palm facing down, and the other downward with the palm facing up. Return to the center and switch directions. Repeat three times.

D: Looking back to cure the five strains and seven injuries: Slowly turn your head to the left as far as possible, without moving your knees, and look over the shoulder. Return to the center and repeat three times on each side.

E: Shaking the Head and Swinging the Tail to Dispel Heart-Fire: Bend your knees to do a lower horse stance. Place your hands on the thighs with the elbows facing out. Swing the head from right to left and your buttocks from left to right before returning to the center. Repeat three times on each side.

F: Clasping fists and glaring to increase strength: Raise both hands above the head. Press them down to the chest level before rotating them behind your back. Bend forward while moving your hands from your back to your toes. Raise your hands again as you straighten up. Repeat six times. Slowly increase your reach each time you bend forward.

G: Clench your fists and glare with anger to increase strength: Bend your knees to do a lower horse stance. Pull both hands in and form fists next to the waist. Punch with your left fist forward, with the eyes facing forward. Then pull your fist back into the waist. Repeat three times on each side.

H: Seven jolts on the back dispel all ailments: Raise your heel and land with a gentle rocking motion. Repeat six times.
