# Supplementary figures and images for: Comparative effects of Baduanjin versus brisk walking on postural control and multidimensional functions in early-to-mid-stage Parkinson's Disease: a randomized controlled trial
Source: Front Neurol. 2026 Apr 22;17:1792257. doi: 10.3389/fneur.2026.1792257 (PMC13143543; doi:10.3389/fneur.2026.1792257)

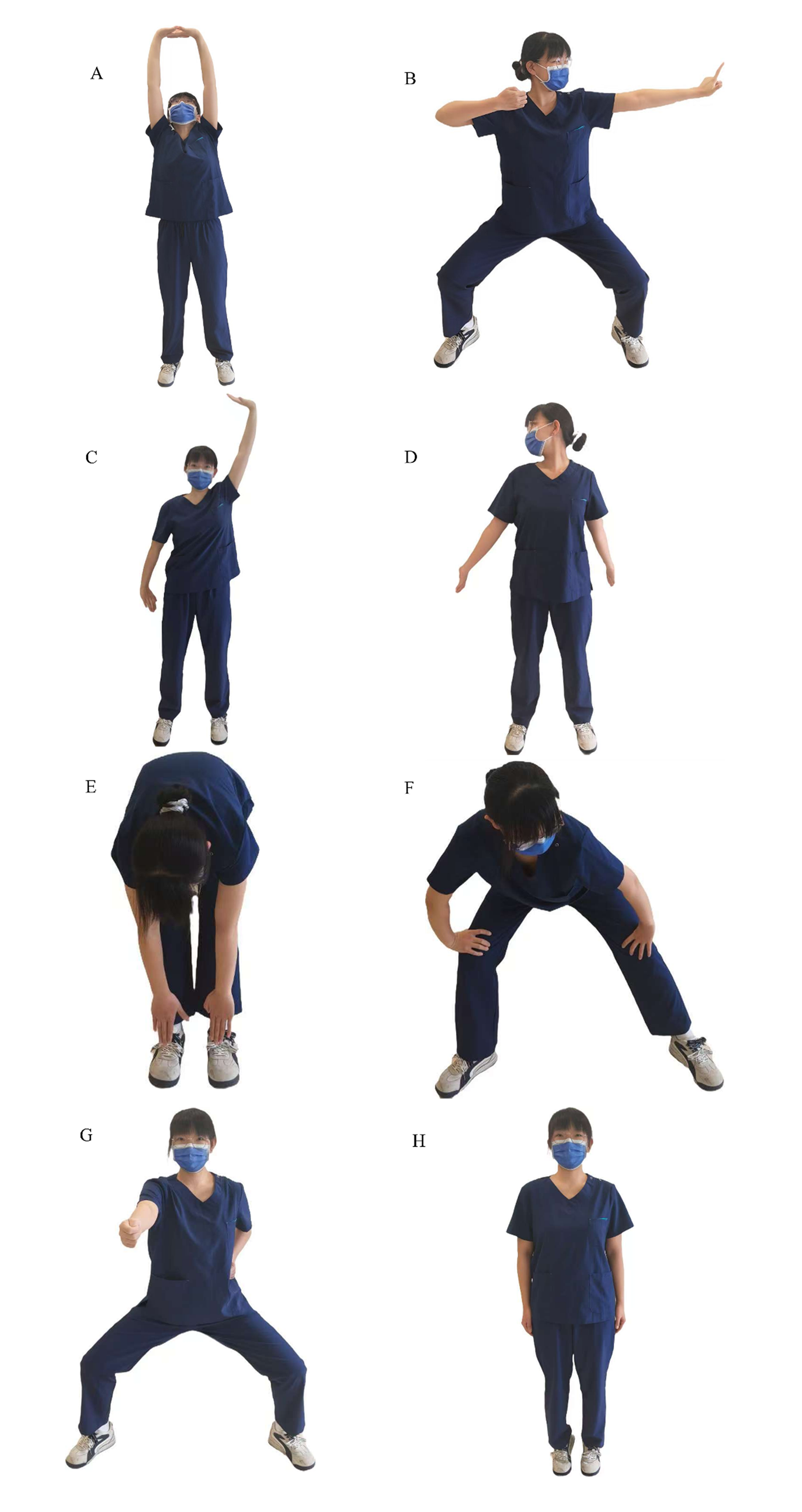

Supplement: Supplementary file 2 [file Image_1.tif]
